# Security Challenges Toward In-Sensor Computing Systems

**DOI:** 10.1145/3716368.3735176

**Published:** 2025-06-29

**Authors:** Mashrafi Kajol, Nishanth Chennagouni, Wei Lu, Qiaoyan Yu

**Affiliations:** Electrical and Computer Engineering, University of New Hampshire, Durham, NH, USA; Electrical and Computer Engineering, University of New Hampshire, Durham, NH, USA; Computer Science, Keene State College, Keene, NH, USA; Electrical and Computer Engineering, University of New Hampshire, Durham, NH, USA

**Keywords:** In-sensor computing, hardware security, fault attack, PDK attack, covert channel attack, privacy

## Abstract

In-Sensor Computing (ISC) systems emerge as a promising alternative to save energy on massive data transmission, analog-to-digital conversion, and ineffective processing. While the new paradigm shift of ISC systems gains increasing attention, the highly compacted systems could incur new challenges from a hardware security perspective. This work conducts a literature review to highlight the research trend of this topic and then performs comprehensive analyses on the root of security challenges. To the best of our knowledge, this is the first work that compares the security challenges of traditional sensor-involved computing systems and emerging ISC systems. We conduct a comprehensive analysis of ISC’s design phases to identify significant vulnerabilities and attack surfaces. Furthermore, new attack scenarios are predicted for board-, chip-, and device-level ISC systems. Three proof-of-concept demos are provided to reveal the consequences of the attacks for three different levels. Our findings emphasize the urgent necessity for sophisticated defense mechanisms and inspire researchers to work on new countermeasure designs against unique hardware security threats in ISC systems.

## 1 Introduction

**Figure 1:**
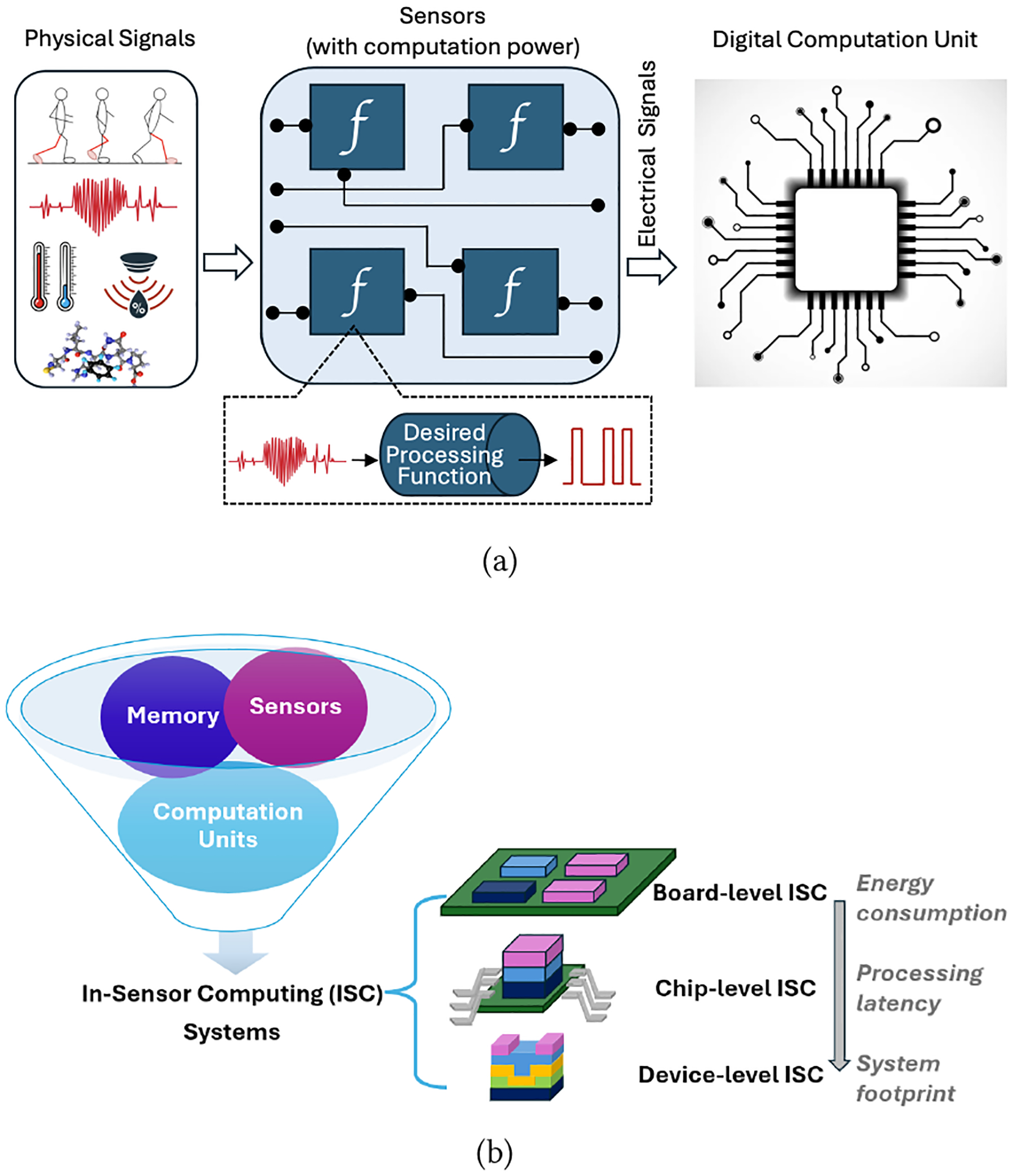
A conceptual diagram for in-sensor computing, (a) overview and (b) technology integration.

Traditionally, massive sensor data are continuously collected to support external processing like Artificial Intelligence (AI)-based analysis and prediction [20], which consumes significant power and introduces delays. To address this limitation, we need a sensing system that is power-efficient, compact, and capable of processing large amounts of data in real time without sacrificing accuracy. One promising direction is to enable sensing units [6] to offer additional computing capability [10, 26] than a simple sensor. In-Sensor Computing (ISC) emerges as a new computation paradigm [23] to address the increasing concern on latency and energy consumption in sensory data transmission, analog-to-digital conversion (ADC), and data pre-processing. Sensing units in an ISC not only sense the target surroundings but also process data at the point of collection, rather than requiring extensive data transfer [23]. A conceptual diagram of in-sensor computing systems is shown in Fig. 1(a). Sensors have some computational power, the essence of which is a desired processing function implemented by leveraging specific material/device mechanisms. Traditional ADC and logic functions are omitted from the data path before the main digital computation unit. The local processing capability ensures real-time monitoring, which is especially critical for health [2, 12], autonomous vehicles [11], and smart surveillance [1] applications.

In-sensor computing systems combine physical phenomena using sensors, process the perceptions, and store them in memory devices. The sensors provide raw analog signals to the computation units for perception, then store them in temporary storage inside a single device. Determining perception before any large computation reduces the workload on post-processing and the physical distance between sensing and computation [10]. Essentially, an ISC system integrates sensors, memory elements, and computation units into one system, as shown in Fig. 1(b). Depending on the integration technology, ISC can be implemented at board, chip, and device levels [23]. As the system footprint decreases from board level to device level, the corresponding energy consumption and analog signal processing latency also decrease due to less ADC conversion and sensory data transmission/receiving.

Existing literature for ISC mainly investigates the materials and device structure to implement desired functions, pursue better performance [9, 16], and examine the feasible integration technologies [10, 11]. While offering significant benefits in performance improvement and power saving [4, 18], ISC could also bring new security challenges. Unfortunately, limited work is available to study new threats and unique attack surfaces for ISC systems. This work fills the gap by making the following contributions:

- The publication trend and the mainstream research focus on in-sensor computing are summarized.
- Comprehensive analyses of the attack scenarios in ISC systems are performed to characterize the new and unique attacks. To the best of our knowledge, this is the first work that analyzes security challenges in ISC systems.
- Proof-of-concept demos are presented to show attack consequences and guide the development of potential countermeasures against hardware security threats in ISC systems.

The rest of this work is organized as follows: Section 2 provides a survey to summarize the state-of-the-art of ISC systems. Section 3 analyzes the proposed attack scenarios at different levels of ISC with demonstrations. Section 4 discusses the potential challenges and future directions. This work is concluded in Section 5.

## 2 Survey of In-Sensor Computing

### 2.1 Prior to In-Sensor Computing

**Figure 2:**
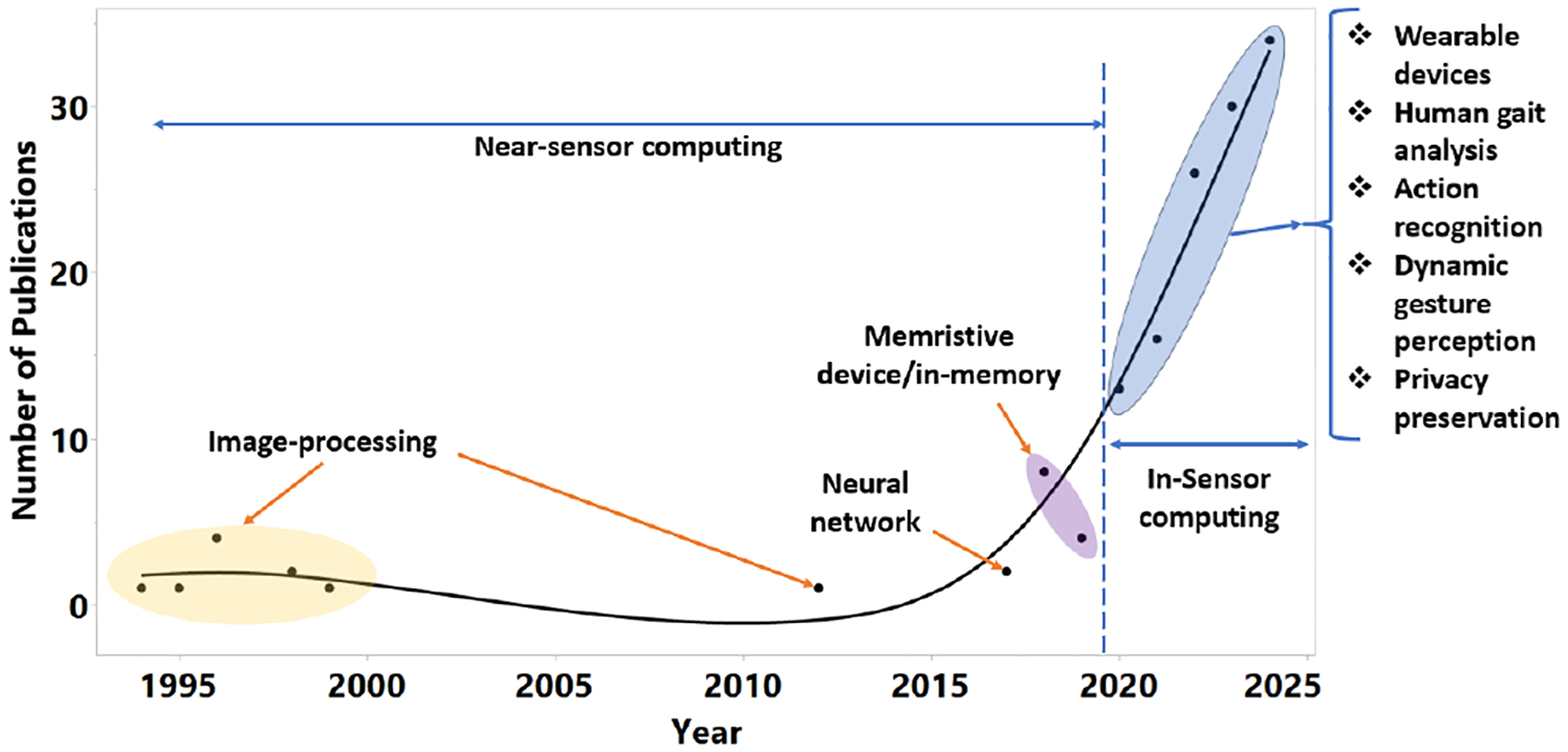
A historical progress of in-sensor computing.

Over the past few years, the field of sensor computing has experienced a noticeable shift. The overall trend from 1994 to 2024 is shown in Fig. 2. The majority of research before 2020 was conducted on near-sensor computing, where computation was performed in close proximity to sensors. There has been an increase in data processing efficiency and a decrease in communication latency between sensors and computing units. As data-intensive applications demand more effective and real-time data processing, there has been a significant rise in publications about ISC after 2020. Ever since then, the popularity of ISC systems has steadily grown because ISC’s advantages in speed, functionality, and energy efficiency are attractive for applications such as wearable devices [14, 24], autonomous systems [7], and AI-driven image recognition [10, 16].

### 2.2 In-Sensor Computing Systems

**Figure 3:**
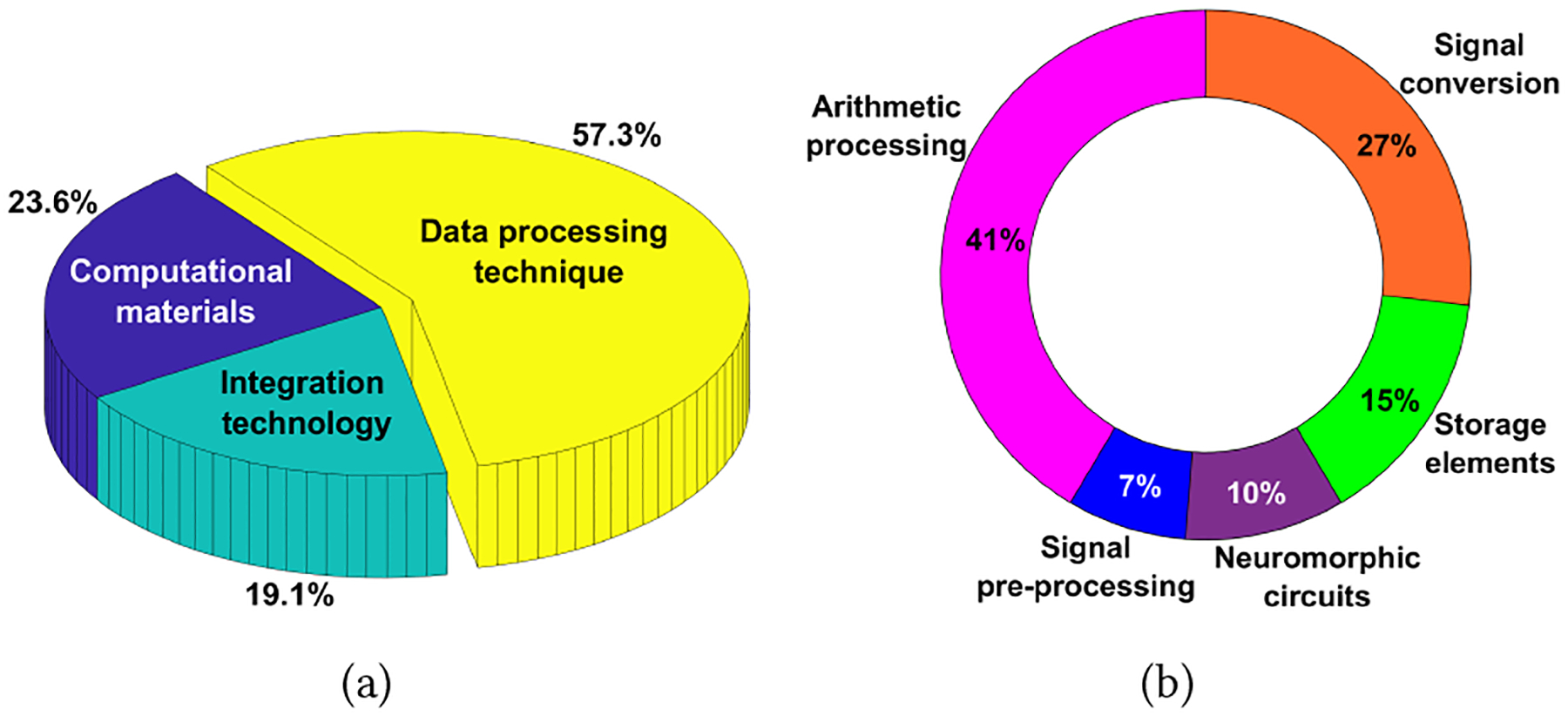
In-sensor computing publications from 2020 to 2024, (a) a pie chart for the percentage of publications in three categories, (b) a donut chart for topics interested in in-sensor data processing techniques.

Based on the underlying focus, we categorize the existing ISC literature into three branches: computational materials, integration technology, and data processing techniques, as shown in Fig. 3(a). The distribution of research articles on ISC indicates that data processing techniques take 57.3% share of the total publications.

**Computational materials-based ISC** leverages novel materials with intrinsic computational capabilities, such as phase-change materials and 2D materials like transition metal dichalcogenides. These materials are designed to sense and process data simultaneously. For example, perovskites-based photoelectric materials [9] can process visual information directly in hardware, eliminating the need for separate computational units.

**Integration technology-based ISC** focuses on how different components (sensors, processors, and memory units) are fabricated and integrated to perform computation at a sensing node. Technologies such as 3D integration of CMOS sensors and memory arrays enable efficient data flow and real-time processing without needing an external processing unit. The 3D stacking of dynamic vision sensors with an in-sensor processing layer [11] helps to compress data and reduce latency in machine vision applications.

**Data processing technique-based ISC** exploits special design and optimization of algorithms and architectures to enable efficient processing of sensor data. For applications such as pattern or image recognition, neuromorphic computing techniques, reservoir computing, and convolutional neural networks (CNNs) have been applied to expand the functionality of sensors. For instance, the work [24] implements reservoir computing in optical sensors to process data for gesture or object recognition with high accuracy and reduced computational complexity. In Fig. 3(b), we zoom in on the research progress made in data processing techniques for ISC systems. The dominant focus areas are arithmetic processing (41%) and signal conversion (27%). Mathematical operations (e.g., multiplication and accumulation) are redesigned for sensors [26]. Signal conversion techniques use new quantization or encoding to transform sensory data into a form that is easier to process or transmit [5]. Other data pre-processing [25] and in-memory computing [21] methods are also interested in the ISC community.

### 2.3 Unexplored Aspect of ISC Systems

**Figure 4:**
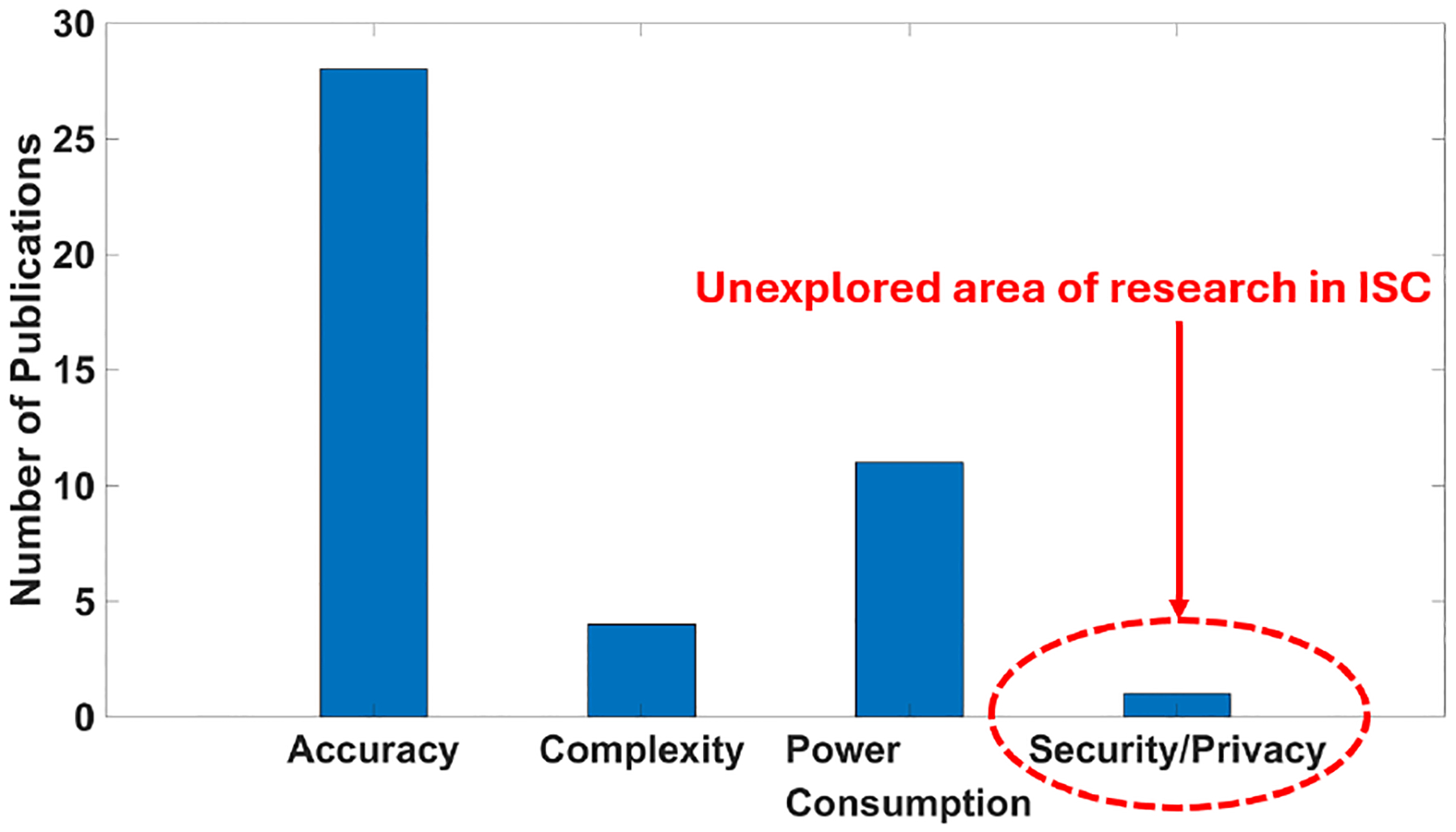
Primary concerns in current ISC literature.

The design metrics discussed in the existing publications for ISC systems include accuracy, complexity, power consumption, and security/privacy. As shown in Fig. 4, most of the existing works [4, 18, 21] pursue high accuracy in data processing. A good amount of work [4, 18] explores power-saving techniques while maintaining accuracy. A few works [14] aim to reduce the complexity of processing units. As the security/privacy aspect is unexplored [7, 22], this work fills this gap by analyzing new attack threats and suggesting potential countermeasure designs.

## 3 Proposed Attack Surface Analyses

### 3.1 Attacks in ISC Design Flow

**Figure 5:**
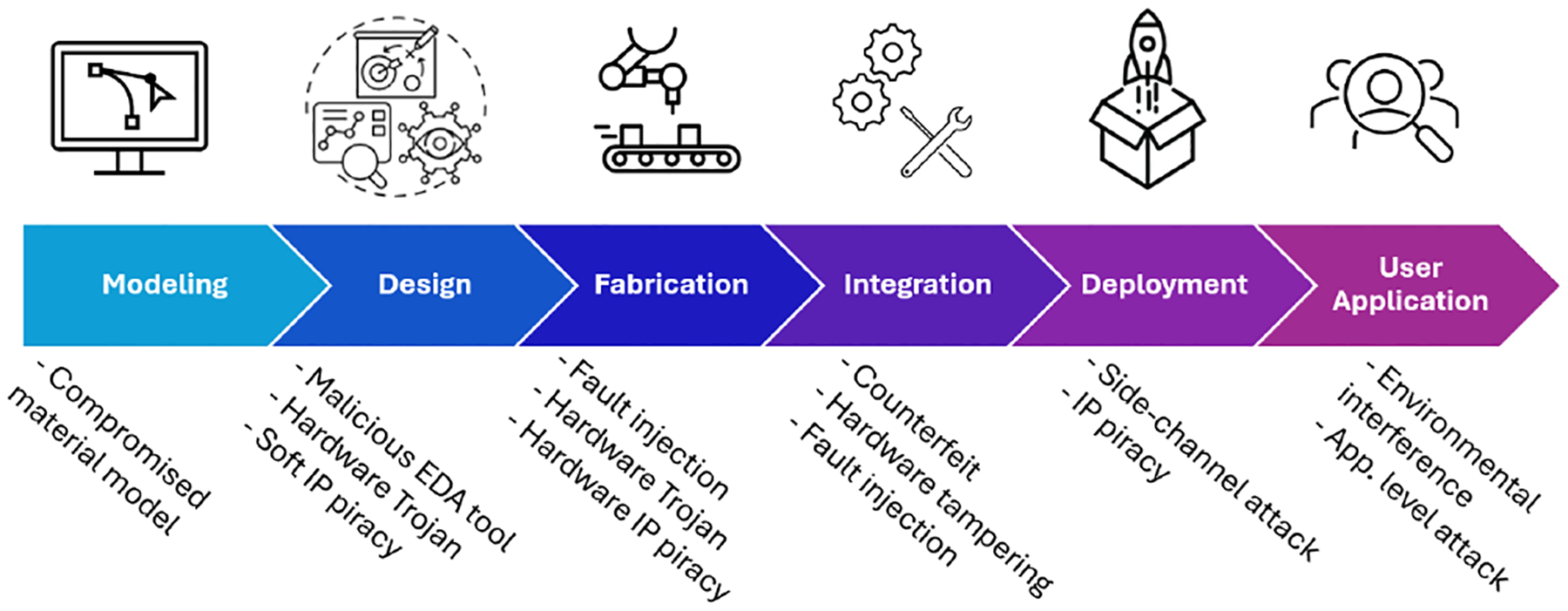
Attacks in different phases of the ISC system’s design and deployment.

Attacks in ISC systems can be performed at every stage of the design flow, as shown in Fig. 5. Since in-sensor computing heavily relies on the special mechanism offered by materials to perform sensing and computation, material modeling plays a vital role in the design flow of ISC systems. As a result, the material and device modeling phase is the first vulnerable stage. Compared to Traditional Sensor-involved Computing (TSC) systems, it is more critical for ISC to ensure the trustworthiness of sensor models and design verification tools. This is because all functionalities, including sensing and computation, of the system are determined and tested at the design phase. The confidentiality of the system design is more centralized in ISC than TSC. In the fabrication stage, a malicious foundry can pose more security threats to ISC systems than TSC systems because the foundry can tamper with both analog and digital components of the system. Typical attacks such as fault attacks, side-channel attacks, and hardware Trojan insertion observed in TSC systems could also challenge the integrity of ISC systems. Table 1 provides more comparisons of different aspects of attacks in TSC and ISC.

### 3.2 Attacks at Device-Level ISC

#### 3.2.1 Proposed New Attacks.

**Figure 6:**
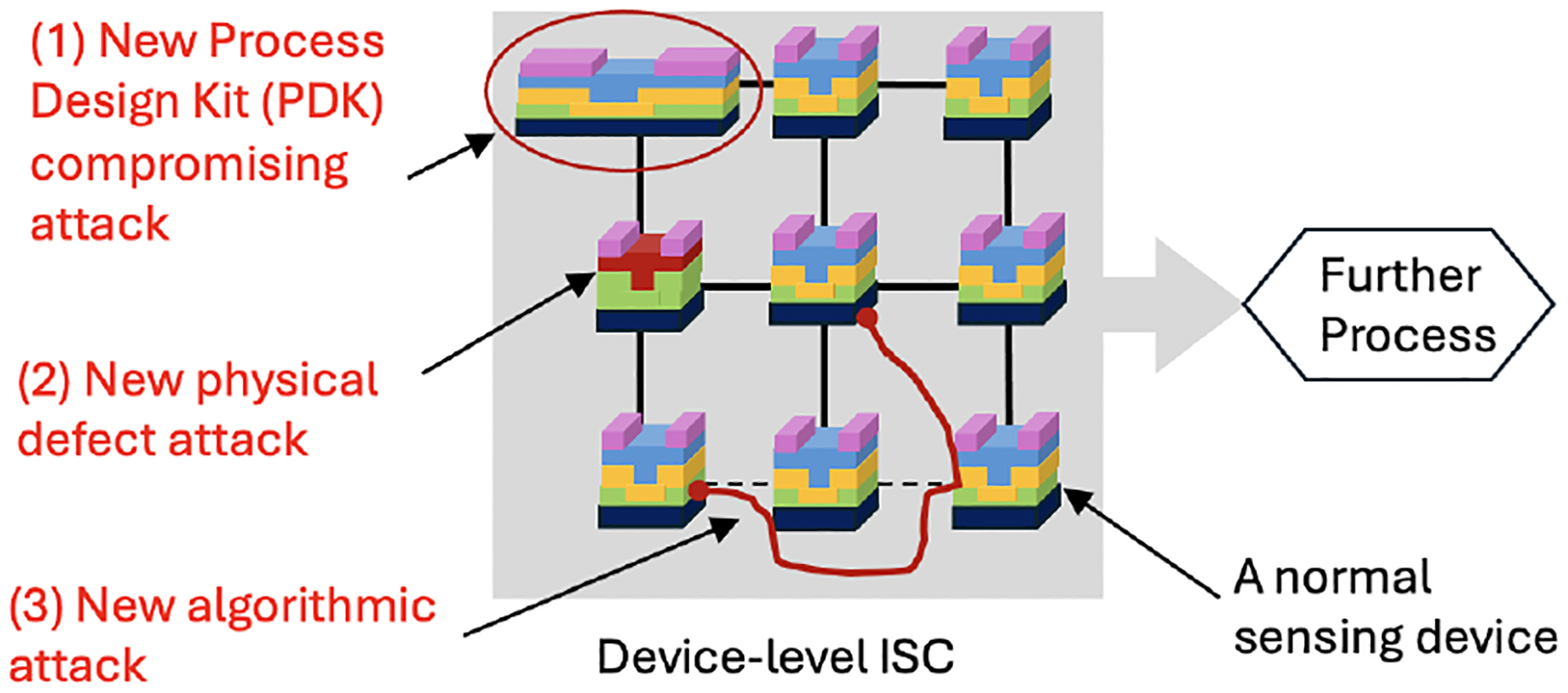
Proposed new attacks at device-level ISC systems.

If all sensing and computation are performed at the device level, as shown in Fig. 6, the implementation of attacks needs more knowledge, advanced tools, and fine-tuning than the attacks at the board- and chip-level ISC systems. Device-level ISC has a higher integration degree than a typical sensor due to the increased computation or processing element. We envision that there will be at least three new attack surfaces in device-level ISC. As all customized processing is tightly coupled with sensing materials, the integrity of each sensor is critical to ensure both functionality and security.

- The first potential attack happens when we incidentally adopt a **compromised process design kit (PDK)** from third-party material providers. Without being aware of the trustworthiness of PDK, we could instantiate a sensing device that carries undesired electrical properties. As a result, the ISC could experience malfunctions.
- The second new attack type is **physical defect attack**. The existing literature [10, 23] shows that an ISC system is often composed of an array of sensing devices. Once the array size becomes large, a small number of defective devices could be overlooked, especially in the absence of exhaustive testing.
- The third new attack is **algorithmic attack**. As the logic function of a device-level ISC heavily relies on the physical mechanism of sensing material [8, 9], we envision that the interconnect network among sensing devices could be tampered with to interrupt the original computation algorithm.

#### 3.2.2 Demonstration of PDK Attack.

**Figure 7:**
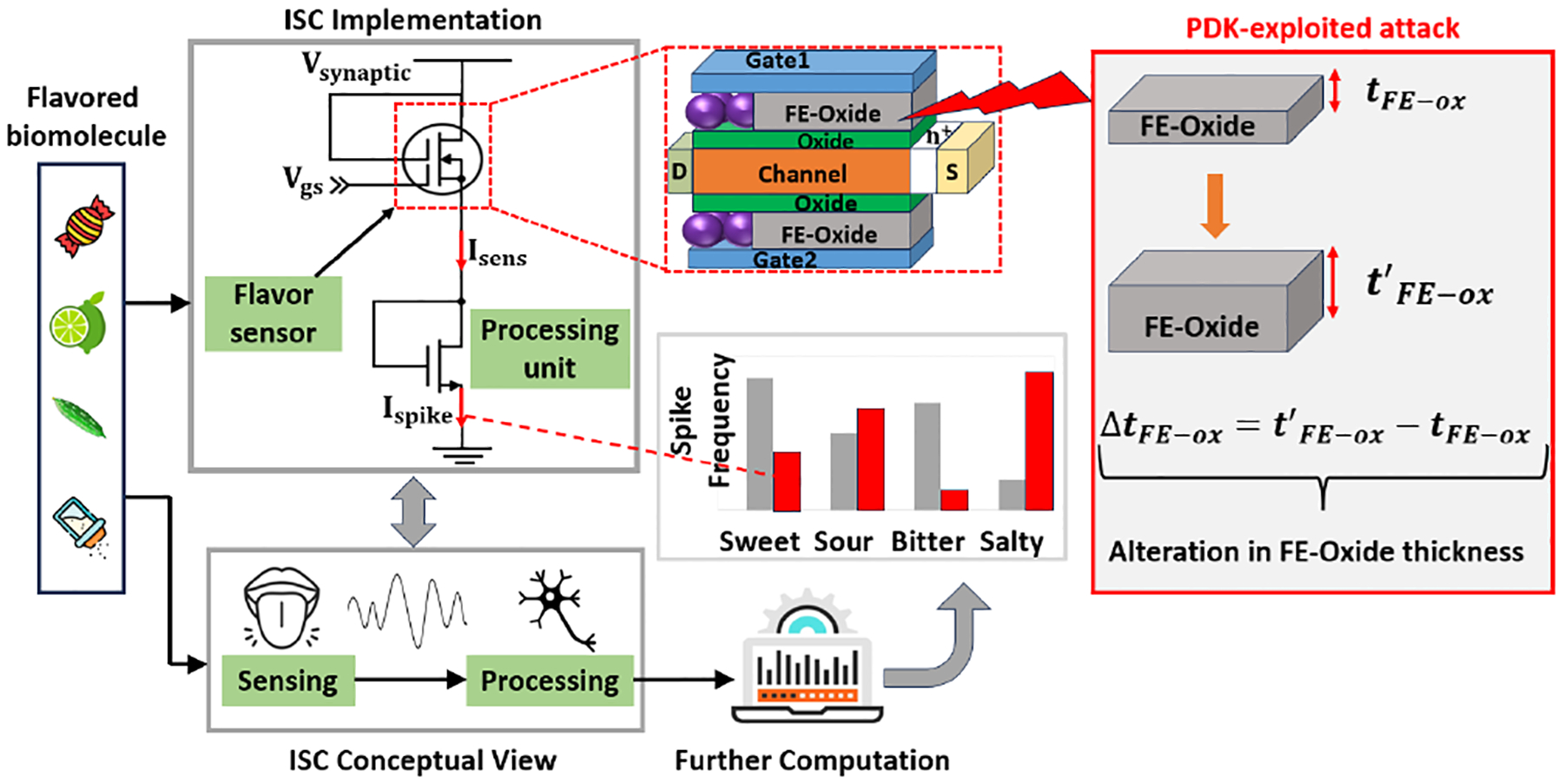
PDK attack on an ISC for biomolecule detection.

An ISC-based gustatory system [8] has the potential to be widely utilized in medical applications and food/beverage industries. The simulation setup is shown in Fig. 7. The ISC system first senses the flavored biomolecules and then preprocesses them for future computation to identify one of the flavors, including sweet, sour, bitter, and salty. The implementation of that ISC is composed of a double-gate ferroelectric tunnel FET (DG-FE-TFET) (i.e., sensor) and a single transistor (i.e., spike analysis circuit). We use this setup to demonstrate how a PDK attack happens at a device-level ISC and the attack impact.

**Figure 8:**
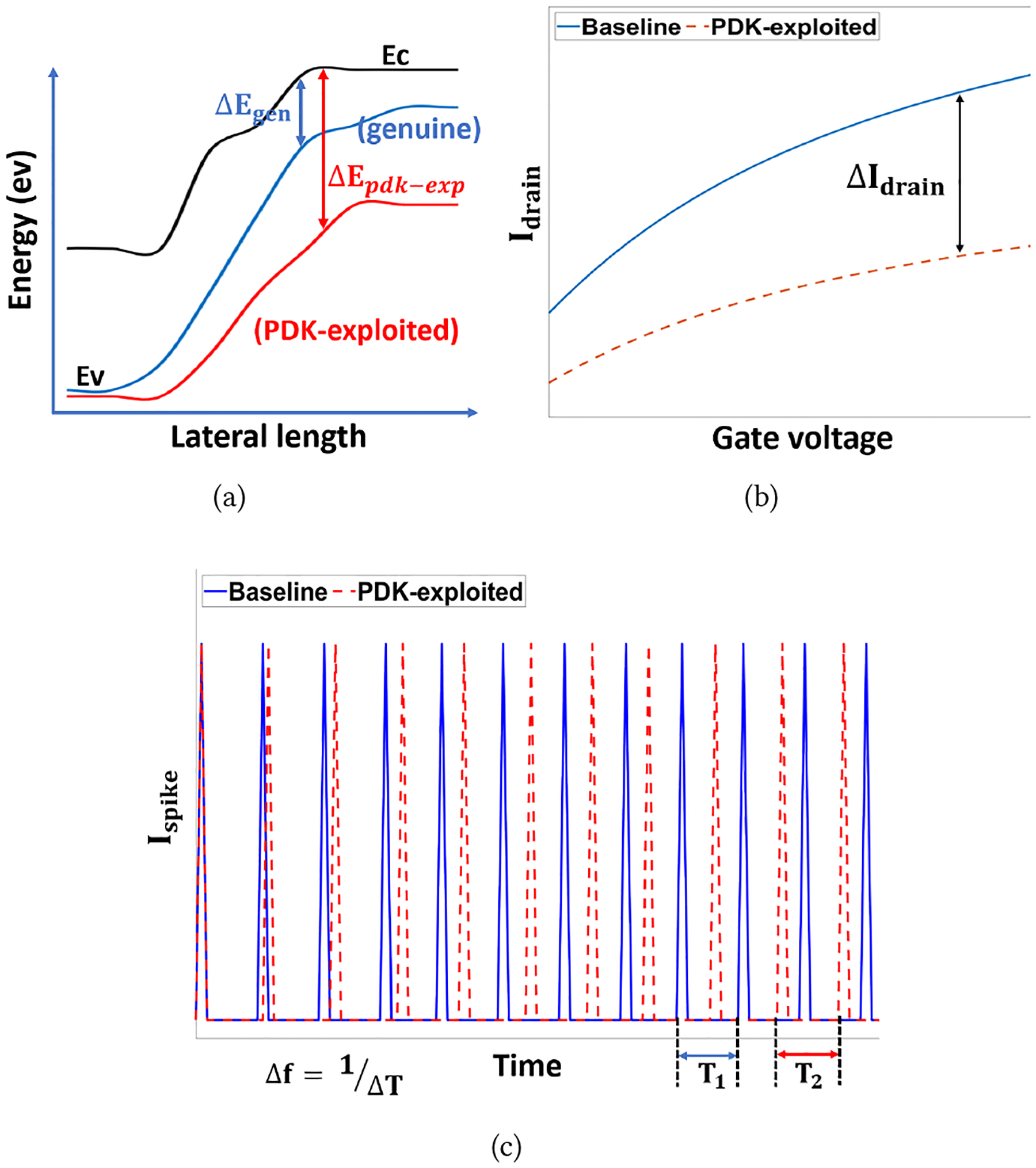
Parameter variation for the compromised device, (a) changes in energy band (conceptual), (b) changes in I-V characteristics, and (c) changes in detection output (frequency).

We assume that the ISC system designer utilizes an untrustworthy process design kit offered by a third-party provider. The single transistor emulating spiking behaviors operates normally, but the transistor acting as a flavor sensor suffers from the PDK attack. Due to the malicious PDK, the energy band of the sensing material is altered by modifying physical parameters. Small changes (FE-Oxide thickness) in the sensing device (DG-FE-TFET) cause a significant variation in the energy band (ΔE_*pdk−exp*_) due to reduced tunneling probability, as shown in Fig. 8(a). The energy band affects the I-V characteristics of the TFET device. The significant change in I-V characteristics, as shown in Fig. 8(b), cannot be captured by using traditional probing. Due to the high integration density and the lack of probing locations, a small change in the FE-Oxide thickness of TFET was not detected until the application completed massive data processing. At the output end, the variation due to the compromised device is shown in Fig. 8(c). The offset frequency caused by the wrong I_spike_ leads to the wrong biomolecule recognition, ultimately resulting in incorrect sensing output after further processing.

##### Lessons learned from this demo:

The PDK-exploited attack on this modeling-based device-level example indicates that (1) ISC systems offer fewer probing opportunities to assist debugging and attack diagnosis at the early stage, in which an attacker could take that opportunity to modify specific devices by exploiting PDK, (2) anomaly detection after the computation stage is more challenging than traditional sensory data because the processed data shows a negligible change, which could be identified as noise.

### 3.3 Attacks at Chip-Level ISC

#### 3.3.1 Proposed New Attacks.

**Figure 9:**
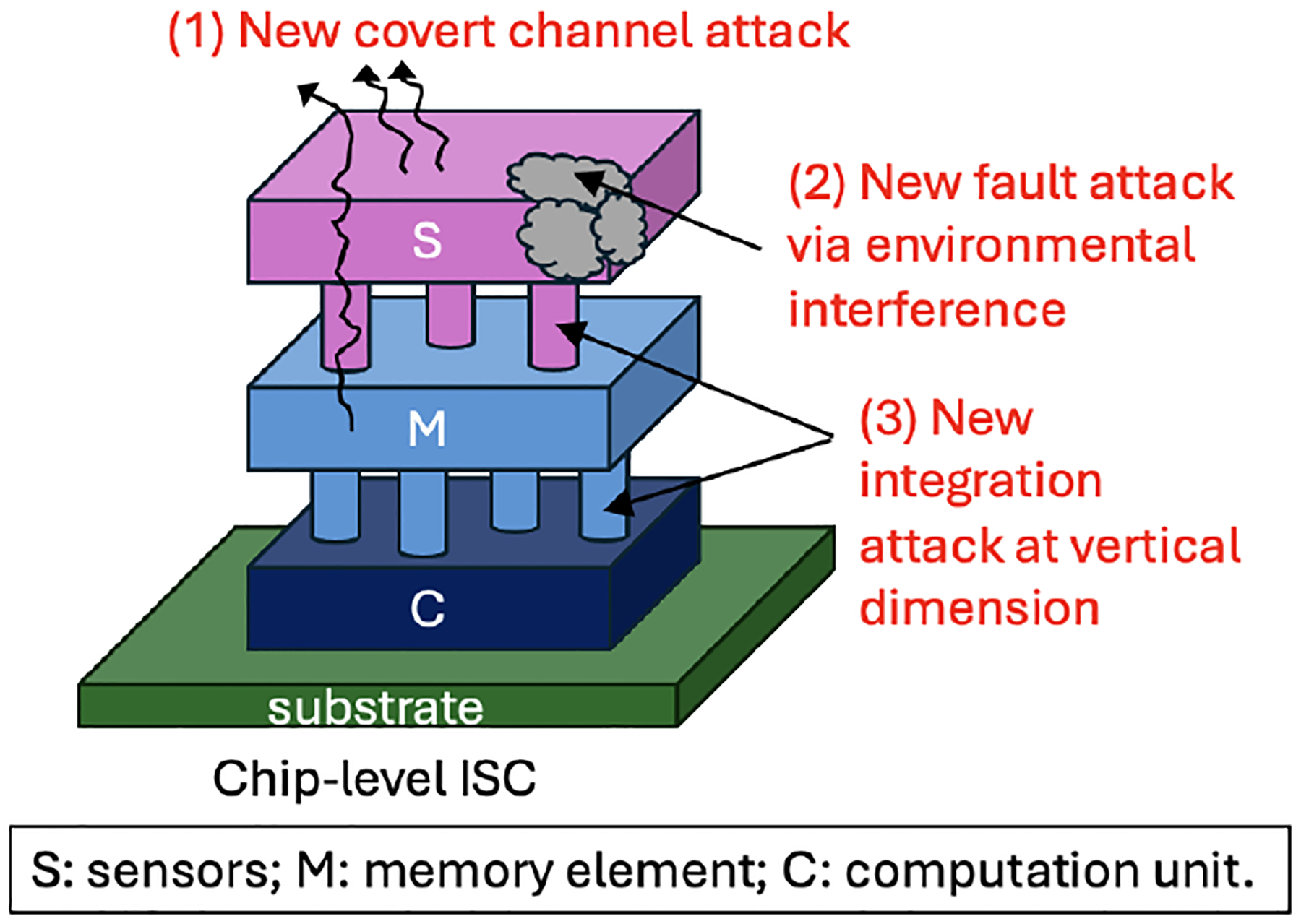
Proposed new attacks at chip-level ISC systems.

Sensor nodes will be deployed to hostile public locations, where sensor devices are vulnerable to physical attacks. We could not completely isolate or shield the systems from the sensing environment; otherwise, the system could not function as it is supposed to. Figure 9 shows a stack-based three-dimensional (3D) ISC system and highlights the unique attacks that could occur in chip-level ISC systems.

- Compared to TSC systems, chip-level ISC could offer more exploration space to establish **covert channels** because of tightly coupled sensing, storage, and computation elements. Sensors could be exploited as a source of information leakage via purposely skewing signals, well-crafted modulation, and electromagnetic signal emission.
- The layers of a chip-level ISC are primarily based on analog circuits, which could suffer from **analog fault attacks** via environmental interference. Since different sensor materials vary dramatically in nature, it is not realistic to generalize the fault attack model for all analog faults in ISC. Thus, the fault attack in ISC should be studied case by case.
- The vertical stack in chip-level ISC makes it vulnerable to **new integration attacks**, particularly the attack targeting the vertical dimension. Unfortunately, it is difficult to examine the integrity of the vertical communication channels during the post-fabrication testing phase. Since a chip-level ISC has a small footprint, there is limited space available for probing. As a result, we may face challenges in ensuring the integrity of the 3D integration process for ISC.

#### 3.3.2 Demonstration of Covert Channel Attack.

**Figure 10:**
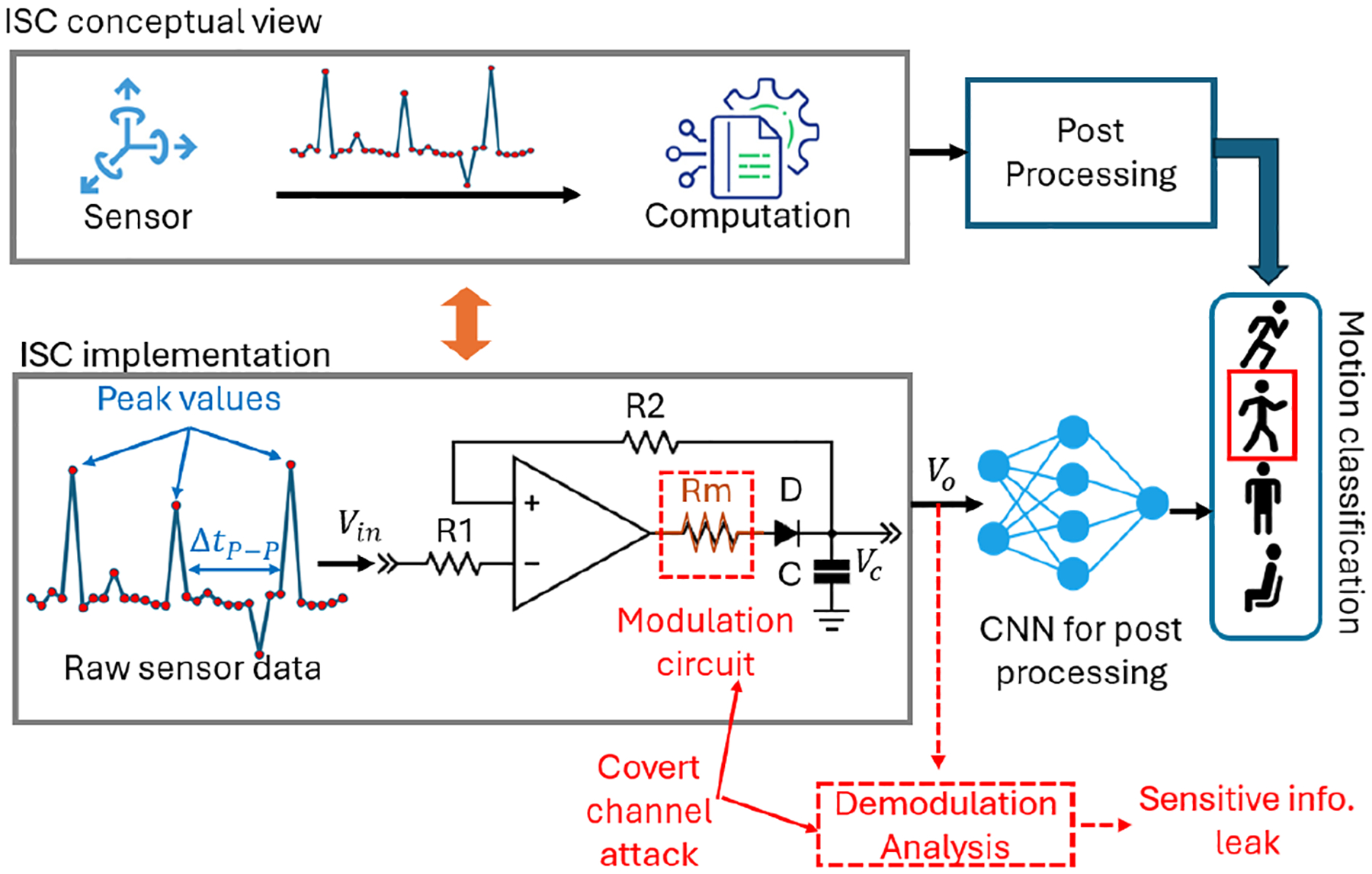
Covert channel attack on a chip-level ISC.

We use a chip-level ISC system shown in Fig. 10 to demonstrate an example of a covert channel attack. This ISC preprocesses the raw sensor data to capture peak values for the convolutional neural network (CNN) that classifies human activities in the later post-processing stage. The raw sensor data is generated by an inertial measurement unit (IMU). The peak values are obtained by a peak detection circuit composed of two resistors *R1* and *R2*, an operational amplifier, a diode *D*, and a capacitor *C* [15]. We consider that the IMU sensor and the peak detection circuit are integrated into a chip.

We assume that the covert channel is implemented during the ISC fabrication stage when the attacker manipulates the fabrication process to introduce extra resistive material such that an equivalent resistor *Rm* will be added before the diode. The *Rm* resistor modulates the sensor data and will change the peak value of the ISC output as described in Equation (1).

(1) $$V_{o}{\left(t\right)}_{\text{modulated}}=\left\{\begin{array}{ll} V_{in}(t); & \left(V_{in}(t)>V_{c}^{\prime }(t)\right)\\ V_{in}(t)*\left(1-e^{\frac{t}{Rm*C}}\right); & \left(V_{in}(t)\leq V_{c}^{\prime }(t)\right) \end{array}\right.$$

**Figure 11:**
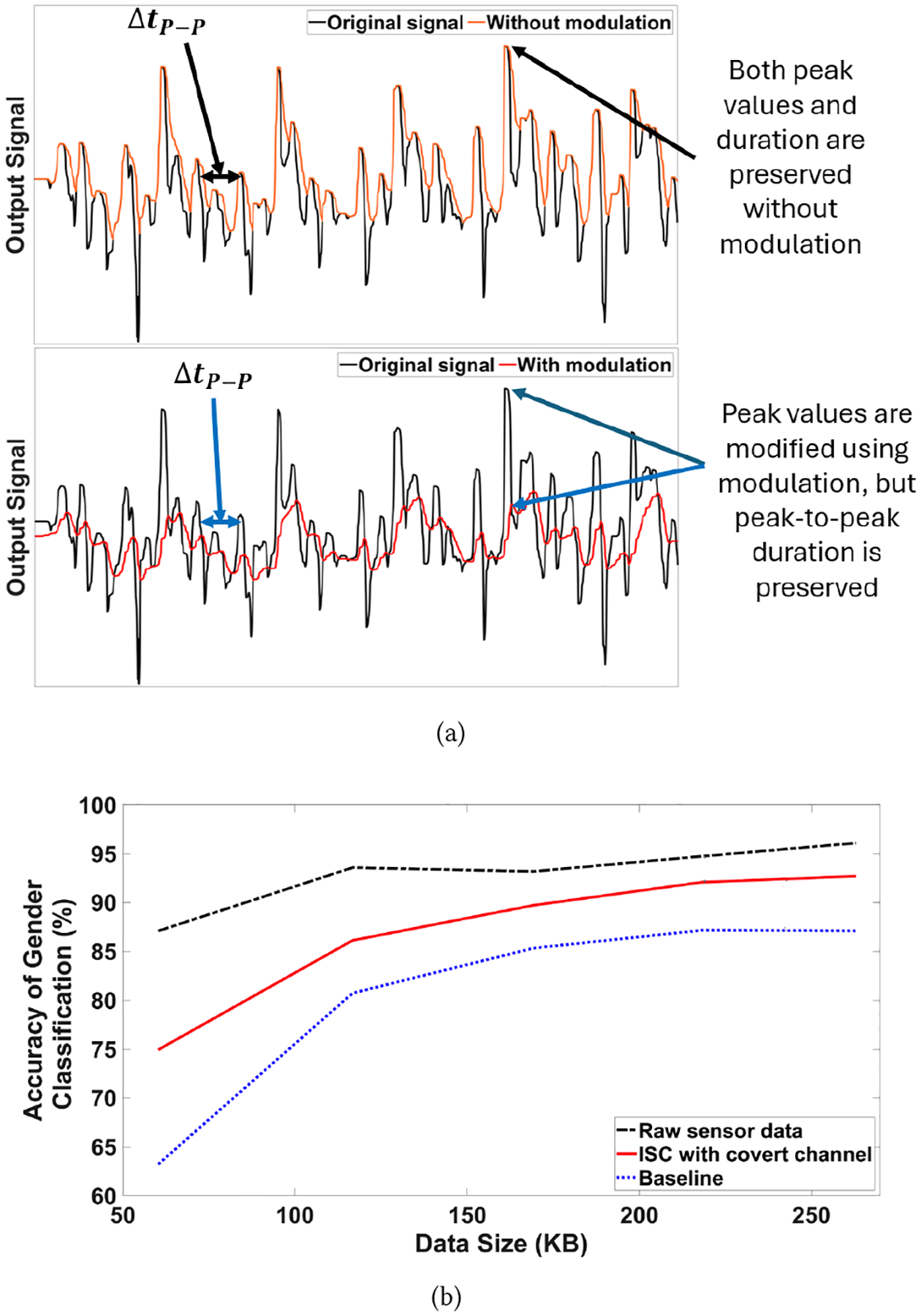
Covert channel attack results, (a) change in output for with and without modulation, (b) accuracy for gender classification.

Where *V*_*in*_ is the sensor raw data and $V_{c}^{\prime }$ is the previous voltage carried by the capacitor. Once the tampered ISC is deployed to an application, the attacker could demodulate the output of the ISC and perform analysis to extract the information of interest. In the following case study, we adopted the raw sensor data from MotionSense [13] and labeled it as *original signal* shown in Fig. 11(a). The outputs of the ISC *without modulation* or *with modulation* (i.e., covert channel attack) are shown in the orange and red curves, respectively, in the two subplots of Fig. 11(a). The top subplot indicates that the computation circuit in the ISC without an attack successfully preserves the peak values and the duration between two peaks. In contrast, the bottom subplot shows that the computation circuit with the attack resistor *Rm* alters the peak values but keeps the same peak-to-peak duration Δ*t*_*P−P*_. This is because the resistor *Rm* increases in the time constant and thus, the output Vo needs more time to reach its original peak. As the raw sensor data changes quickly, the original peak value cannot be obtained exactly. However, by carefully choosing *Rm*, it is still possible to preserve the same time interval between peaks.

To measure the success rate of the covert channel attack that aims to retrieve private information (gender) from the motion sensor data [13], we compared the accuracy of gender classification achieved by a CNN in three cases: raw sensor data, ISC with a *Rm*-based covert channel, and ISC without covert channel (i.e., baseline). As shown in Fig. 11(b), the covert channel attack enables the compromised ISC to achieve 14.8% more accuracy (on average) in gender classification than the baseline. When more ISC data are fed to CNN for post-processing, the accuracy of gender classification for ISC with a covert channel is only 2.8% less than that for the raw sensor data. This example demonstrates the feasibility of implementing a covert channel attack in the chip-level ISC and the consequence could be significant.

##### Lessons learned from this demo:

The covert channel attack in integrated chip-level ISC signifies that (1) the chip-level ISC can create a good path to host a covert channel, where limited sensitive data leaks can reveal sensitive information with high accuracy, (2) the integration of sensing and analog computation units makes ISC difficult to find and isolate covert channel modulation paths using traditional security countermeasures.

### 3.4 Attacks at Board-Level ISC

#### 3.4.1 Proposed New Attacks.

**Figure 12:**
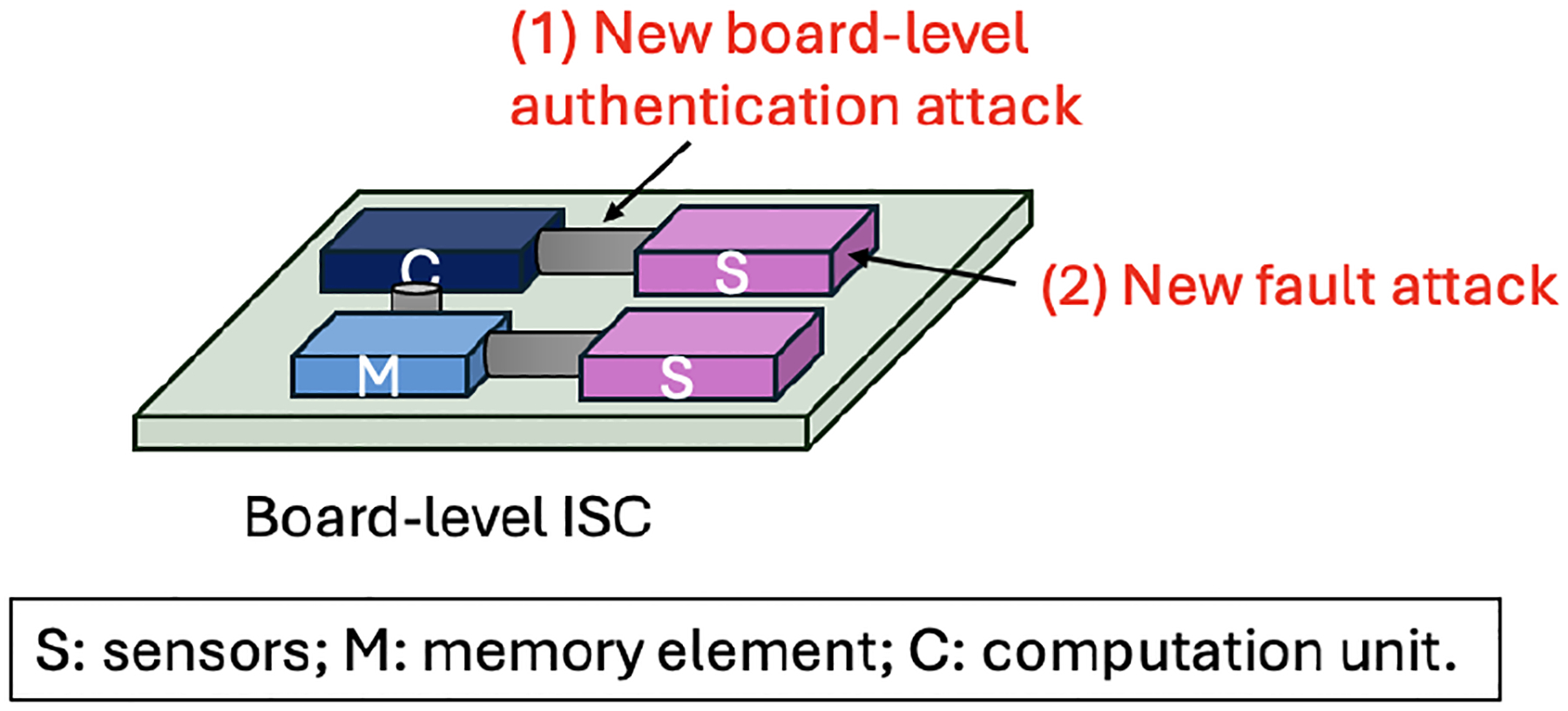
Proposed new attacks at board-level ISC systems.

The board-level ISC systems are vulnerable to both digital and unique analog attacks. The digital memory elements and computation units in a board-level ISC will be vulnerable to the same attacks as we see in traditional sensing systems. Due to the same reasons that we mentioned in Section 3.3.1, board-level ISC could be challenged by covert channel attacks, too. Moreover, we should prepare for new attacks highlighted in Fig. 12.

- In board-level ISC, sensing units on the board directly provide analog reading to the computation unit, so there might be a lack of device authentication. The interconnect between the computation unit and sensors will be vulnerable to **authentication attacks**. Due to the analog nature of sensors, existing digital authentication methods could not work properly in this scenario. We envision that analog authentication will be valuable in this case.
- New **fault injection attacks** that exploit the sensor’s physical/chemical mechanism will challenge the integrity of sensor readings. The existing fault attacks on digital components are easy to detect thanks to the precise digital specification. In contrast, ISC performs analog computing; thus, it is difficult to have a quantitative description of abnormal behaviors.

#### 3.4.2 Demonstration of Fault Injection Attack.

**Figure 13:**
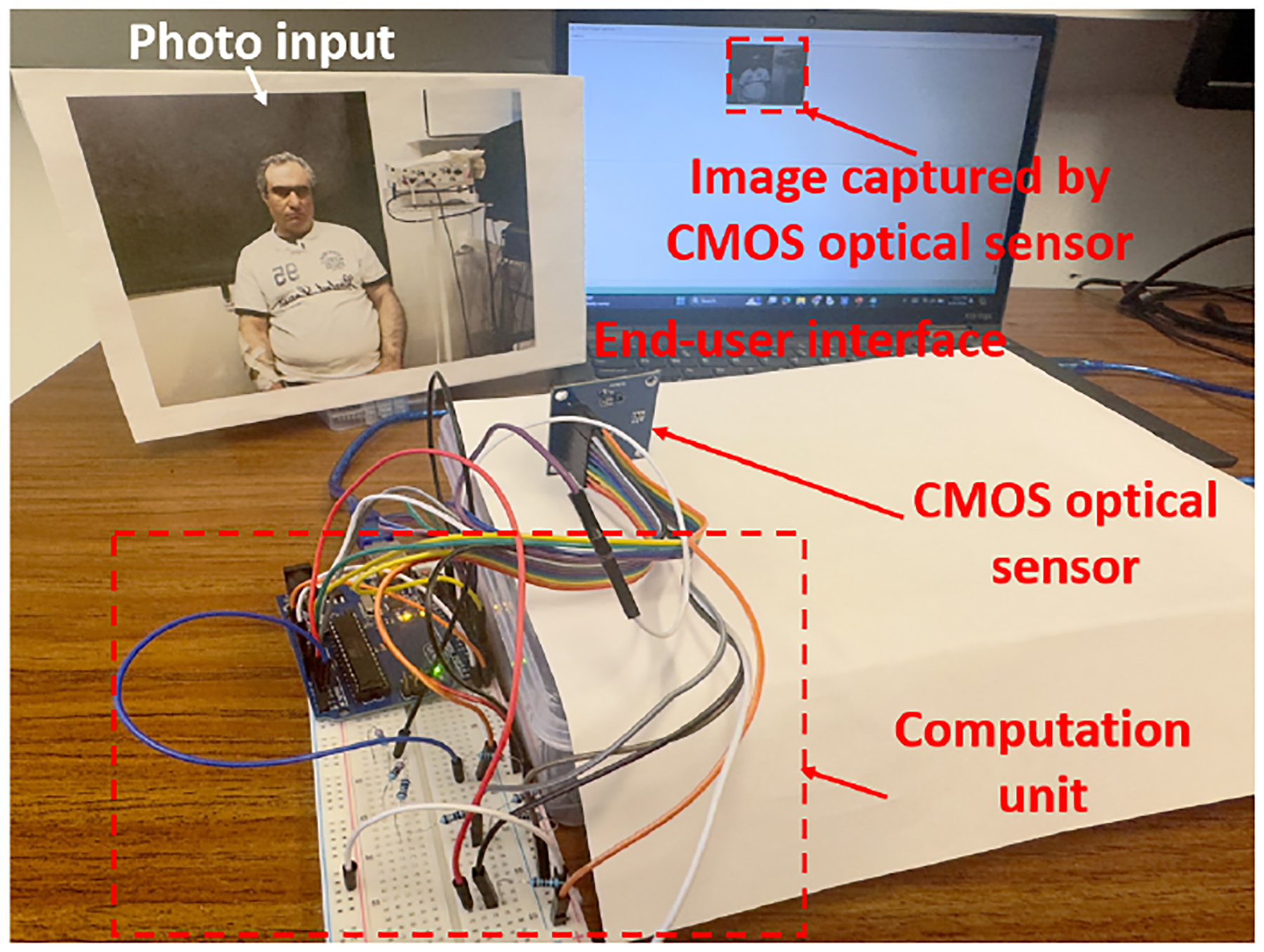
Experimental setup of an ISC for expression detection.

We use a proof-of-concept example to demonstrate that a board-level ISC system is vulnerable to a low-cost fault injection attack via environmental interference. This ISC system captures a facial expression image via a CMOS optical sensor [17] and then utilizes the Py-Feat machine learning model [3] to classify human expressions. We integrated the optical sensor with a microcontroller-based computing unit. The computing unit receives serial data and processes it to detect different facial expressions. The physical experimental setup is shown in Fig. 13.

**Figure 14:**
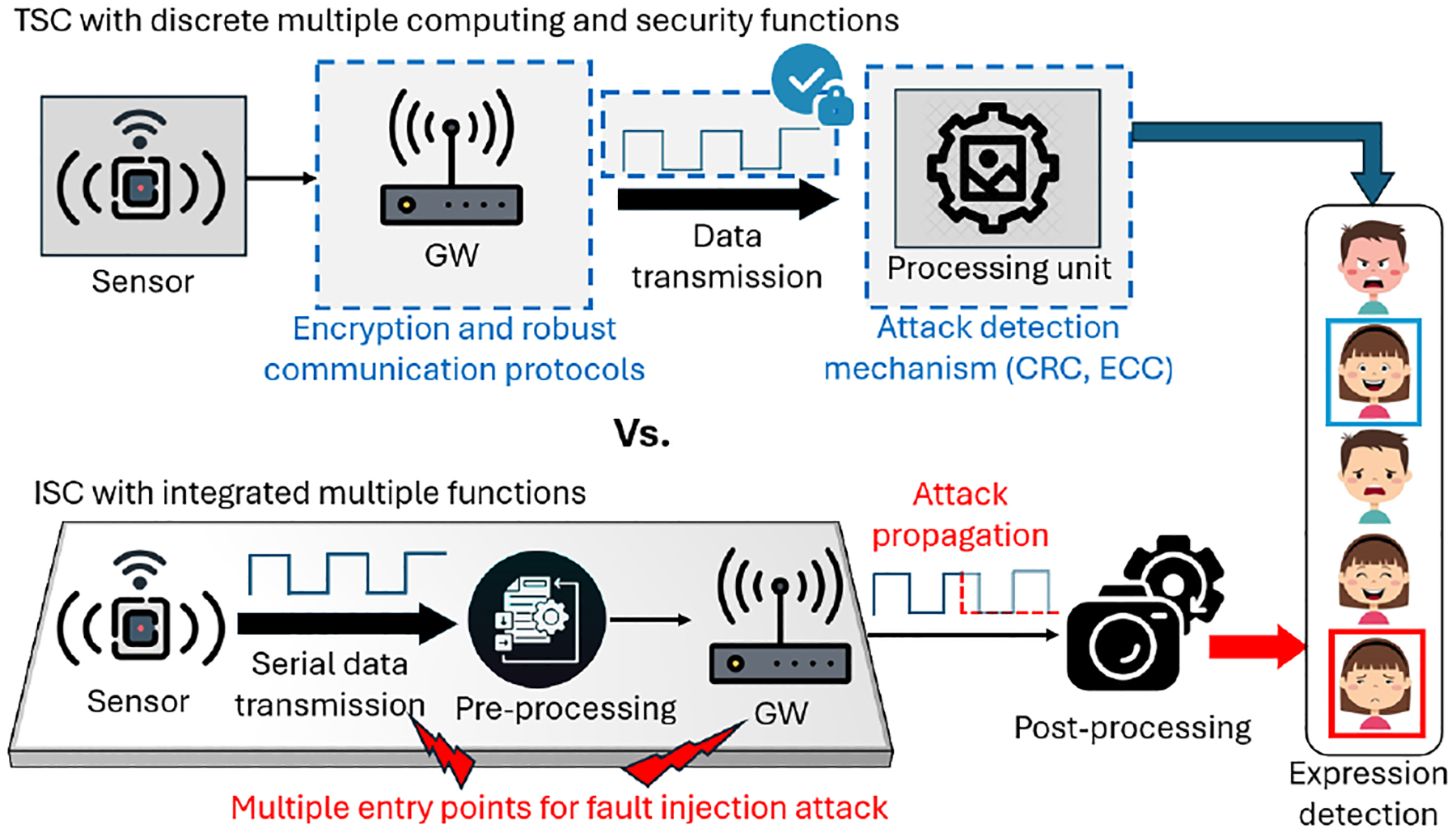
Comparison between the conceptual views of the TSC and board-level ISC for our proof-of-concept example.

**Figure 15:**
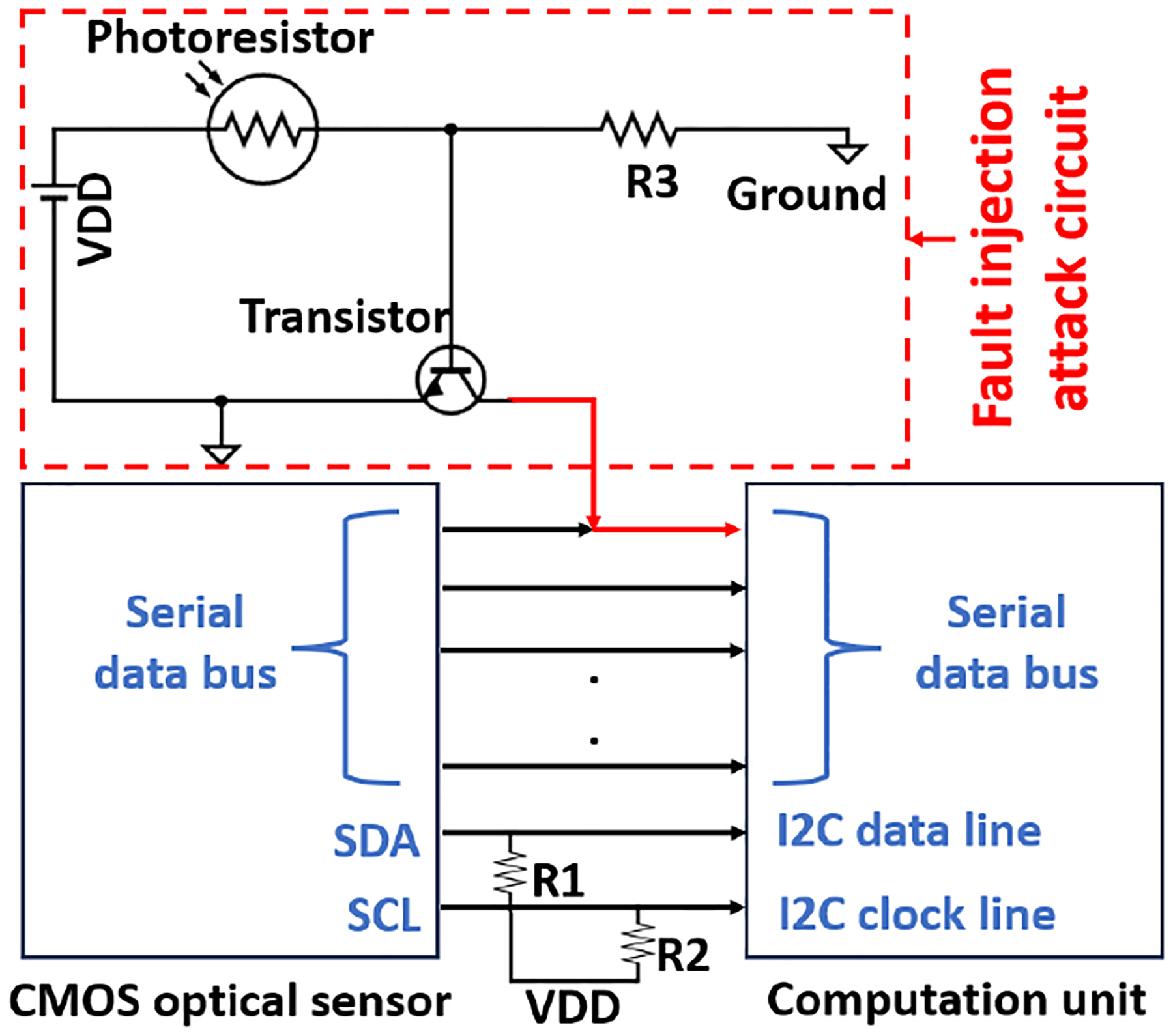
Fault injection attack on an optical sensor in the ISC system.

We assume that an attacker knows only the overall function of the ISC system but lacks knowledge of the ISC implementation details. Given that it is a board-level ISC system, it is reasonable to further assume that the attacker could locate the board-level interconnection between the optical sensor and the computation unit. The comparison between TSC and ISC is shown in Fig. 14. In a TSC system, the sensor and the processing units are utilized as discrete components that communicate through trusted interfaces with distinct security features, ensuring data integrity. In contrast, the ISC integrates multiple units onto a single board, exposing the interconnections (shared resources) between them to the attackers. These interconnections create multiple entry points for fault injection attacks. Attacking a single entry point allows the attack to spread throughout the entire system, as it lacks distinct security layers. Thus, an attacker can inject faults without having any security issues by utilizing malicious circuits in the ISC. To prove this concept, a low-cost malicious circuit composed of a photoresistor, a transistor, and a regular resistor was mounted on the serial data bus between the sensor and the computation unit. The schematic of the malicious circuit and the optical sensor system is shown in Fig. 15. During the system integration stage, the malicious circuit is mounted on the serial data bus. An illumination-adjustable light was used to influence the resistance of the photoresistor and thus sabotage the integrity of the captured images. When a high-intensity light is applied to the photoresistor, the voltage on the transistor’s base terminal is high enough to turn on the transistor and thus pull down the serial data transmission bus, regardless of what sensory data is being transmitted to the computation unit.

**Figure 16:**
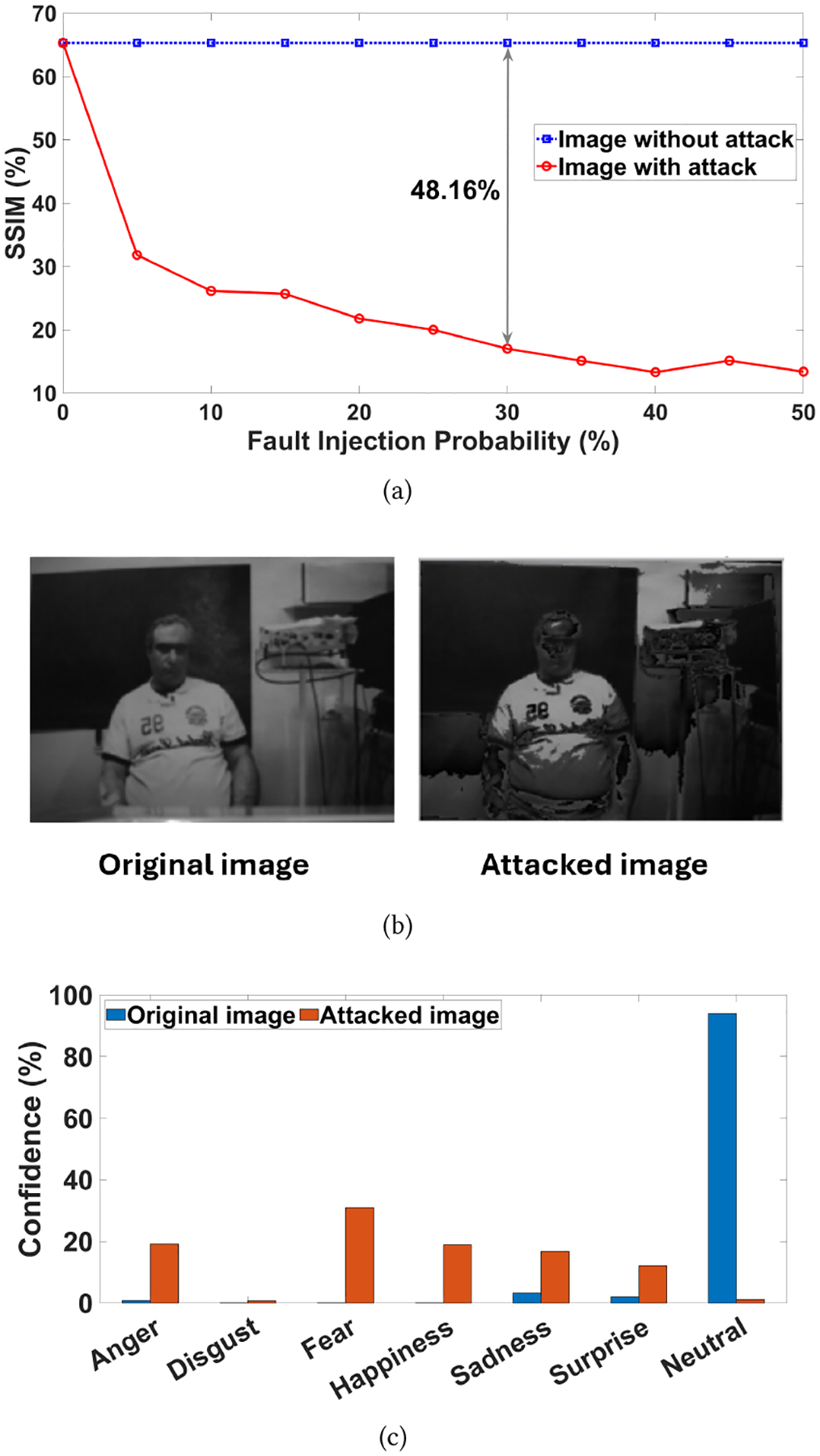
Difference between original and attacked images, (a) SSIM metric comparison, (b) output images at the user interface, and (c) different expression detection.

Since this fault injection attack affects the ISC, we vary the fault injection probability to show the effect of attack propagation through the system. We analyze the propagated attack by comparing the output images, which include those with and without the attack. We utilize an image quality metric—Structural Similarity Index Measure (SSIM) [19]—to compare the structural similarity between the output images and the original image, which is shown in Fig. 16(a). The image without attacks shows constant SSIM over different fault injection probabilities because there is no attack propagated toward the final processing. However, the ISC exhibits continuous degradation as the likelihood of fault injection increases. The reason for this degradation is the fault attack indicated in Fig. 15. The fault attack has transformed some pixel data packets into attack-induced data, which has spread throughout the computation unit. This attack is difficult to detect due to less opportunities to implement security protocols before transmitting the data. Thus, the original image was darkened significantly, as shown in Fig. 16(b). When the image being tampered with goes to the computation unit, the confidence in expression classification is jeopardized. The confidence for different expression detection is shown in Fig. 16(c). The conclusion of a natural facial expression was mistaken as anger, fear, happiness, sadness, and surprise.

##### Lessons learned from this demo:

This example warns us that (1) even though ISC systems have a higher integration degree than TSC systems, attackers can still implement black-box attacks with a low-cost method, (2) since sensors in ISC systems provide more functions than the TSC systems, sensors’ resilience against fault injection attacks will be increasingly important than before, (3) due to lack of authentication between sensors and computation units in the emerging ISC, verification of data integrity should be vital in both integration and deployment stages.

## 4 Challenges and Future Directions

Although ISC systems address the bottleneck in communication from sensors to computation units, more analog signals are involved in ISC systems than in TSC systems. Consequently, existing countermeasures designed for digital systems may not be effective in thwarting new fault attacks, which could be disguised as analog noise. The high integration density of ISC systems makes it more difficult to probe intermediate checking points for early attack diagnosis. Moreover, it is not feasible to authenticate individual sensing components and computation units. As ISC systems exploit various materials for different sensing purposes, it is challenging to develop generic design validation tools to build systems’ equivalent models and examine the integrity of system design and implementation. The inherent nature of parallelism in some ISCs (i.e., image processing, memristive array) demands a new screening process to check all ISC elements. To address those new challenges, research efforts are required in various sectors to maintain data integrity and develop lightweight encryption algorithms tailored for analog processing. Moreover, a hybrid countermeasure may be useful to combine small external validation and robust internal defense. Here are some suggested countermeasure techniques:

- The ISC at the device level needs defenses at several stages, including characterizing the material, checking the integrity of the fabrication, and building a robust design architecture. ISC design kits require sophisticated tools like scanning electron microscopy (SEM) to verify the material characteristics. Wafer-level testing might be useful for fabrication integrity verification. In addition, the distribution of PDK should incorporate cryptographic algorithms to ensure its integrity.
- Unique fault attacks in chip-level ISCs demand more sophisticated noise analysis. Deep-learning tools could be leveraged to automate the attack analysis. Real-time analog signal monitoring (e.g., impedance checking) may provide critical information to detect hardware modification due to a covert channel.
- At board-level ISCs, analog authentication will be beneficial for minimizing the delay and hardware cost. If we could authenticate the sensor component in the analog domain, digital conversion could be omitted. Another promising direction is analog neural networks for real-time attack detection.

## 5 Conclusion

In-sensor computing systems present a promising solution for diverse applications in various sectors to achieve high processing speed and low energy cost. However, hardware security challenges of ISC systems have not been widely investigated, according to the survey. This work fills this gap by analyzing security threats from three different levels based on their architectural characteristics. Our research suggests that ISC may pose potential security concerns due to its inherent close integration of sensing and processing capabilities. Moreover, the traditional countermeasure cannot detect or isolate the new attacks due to fewer debugging opportunities. The attack demonstrations highlight the attack consequences and difficulties in thwarting the attacks, which require expertise in particular systems and sophisticated tools. We hope that our proof-of-concept demos will inspire researchers to make efforts to strengthen the security of ISC systems.

## Figures and Tables

**Table 1:** Comparison of Attacks in TSC and ISC Systems

Systems	Traditional Sensor-involved Computing	In-sensor Computing
Accessibility	**High:** through interfaces between sensors and computation units or remote access	**Limited:** physical access to the entire system, not directly connection to individual components
Prior Knowledge	Require **partial** knowledge of target components	Require **full** knowledge of entire system
Attack Tools	**Low:** many existing tools available to automate attacks	**High:** customized tools are needed
Attack Detection	**Easy:** probe via multiple wire/wireless interfaces and use existing validation tool	**Complicate:** lack generic validation tools and need to localize/differentiate attack sources

## CCS Concepts

• **Hardware → Sensor devices and computing platforms**; • **Security and privacy →** *Hardware attacks and countermeasures*.
